# TiO_2_ Polyamide Thin Film Nanocomposite Reverses Osmosis Membrane for Water Desalination

**DOI:** 10.3390/membranes8030066

**Published:** 2018-08-17

**Authors:** Ahmed Al Mayyahi

**Affiliations:** Department of Chemical Engineering, University of Missouri, Columbia, MO 65211, USA; aaakz5@mail.missouri.edu or sumrabm@yahoo.com

**Keywords:** nanoparticles (NPs), thin film nanocomposite (TFN), reverse osmosis (RO), interfacial polymerization (IP)

## Abstract

In this study, TiO_2_ nanoparticles were inserted into the polyamide layer of traditional thin film composite membrane. The nanoparticles were dispersed in a trimesoyl chloride-hexane solution before interfacial polymerization with *m*-phenylenediamine-aqueous solution. Membrane characterization was performed via contact angle measurements, atomic force microscopy (AFM), scanning electron microscopy (SEM), and water flux, salt rejection, and fouling resistance evaluation. The results indicate that TiO_2_ could effectively improve membrane performance. Water flux increased from 40 to 65 L/m² h by increasing NPs concentration from 0 to 0.1 wt. %, while NaCl rejection was above 96%. Moreover, the modified membrane demonstrated better organic fouling resistance and robust antibacterial efficiency.

## 1. Introduction

Water scarcity is one of the tremendous obstacles facing modern society [1,2]. In the last century, as the world population increased fourfold, the global demand for water septupled. In the next 10 years, many countries are expected to face harsh water crisis; water deficit is not only a naturally caused problem, but also a man-made problem requiring technical solutions [3].

Sea water desalination is an important approach used to supply suitable water for human needs and domestic usage [4]. Reverse osmosis (RO) has become the most widely used desalination technique because of its low cost and simplicity, in contrast with other water treatment approaches [5]. Polymeric membranes are widely used in RO desalination because of their high flexibility, their pore forming mechanism, and their low cost [6].

Among various desalination membranes, thin film composites (TFCs) are considered to be the most efficient [7]. These are usually prepared by the interfacial polymerization (IP) of *m*-phenylenediamine (MPD) and trimesoyl chloride (TMC) on a porous support, typically Polysulfone (PSU) [8]. In 2007, a high-quality polymeric membrane was synthesized by blending nanoparticles (NPs) into the PA layer of traditional TFC. For instance, the membrane embedded with zeolite -NaA NPs showed higher water flux in comparison with the virgin one [9]. In addition to zeolite, different nanoparticles, including carbon nanotubes (CNTs) [10], silica [11], clay [12], and grapheme oxide (GO) [13] were used to modify TFC membranes. All the modified membranes showed improved water flux and high salt rejection. However, thin film nanocomposite (TFN) membranes have a significant disadvantage because of membrane fouling. Membrane fouling leads to water flux declination, which as a result increases operating costs and reduces membrane lifetime [14,15]. To overcome this challenge, Kim and Deng [16] dispersed mesoporous carbons (OMCs) as non-fillers in the PA to reduce the accumulation of bovine serum albumin on the membrane surface. The results indicated that increasing the concentration of OMCs eliminated foulant adsorption. Another study by Ali et al. [17] successfully mitigated humic acid fouling through the incorporation of GO. Recently, Jin et al. [18] prepared TFN membranes with high resistance against *E. coli* bacteria by using silver nanoparticles.

Semiconducting nanomaterials are increasingly used in water purification due to their capability to destroy organic contaminants in waste water [19]. It was claimed that applying UV light on the semiconductors generates holes and electrons on the surface. These holes/electrons could either recombine or interact with organic molecules and as a result reduce organic pollutant content [20,21]. Integrating photocatalytic oxidation and membrane filtration has been demonstrated to be a “win win” approach for reducing the adsorption of fouling on membrane surface [22,23]. Damodar and his coworkers [24] investigated the photocatalytic characteristics of TiO_2_-entrapped PVDF membrane. The obtained results indicated that membrane permeability could be improved upon the addition of TiO_2_, and the modified membrane exhibited better fouling resistance under UV light. In addition, TiO_2_ showed promise for application in water disinfection due to its robust activity in microorganism destruction.

Thus, herein, TiO_2_ NPs were used to produce PA-TFN-PSU membrane with enhanced fouling resistance and high wettability, while maintain the salt rejection.

## 2. Materials and Methods

### 2.1. Materials

Polysulfone (PSU, *M_W_* = 35,000) and *N*,*N*-dimethylformamide (DMF, 99.8%) were obtained from Sigma-Aldrich, St. Louis, MO, USA and used in PSU support fabrication. *m*-Phenylenediamine (MPD, ≥99%, Sigma-Aldrich, St. Louis, MO, USA) and trimesoyl chloride (TMC, ≥98.5%, Sigma-Aldrich, St. Louis, MO, USA) were used as raw materials to synthesis the PA film. TiO_2_ nanoparticles (<100 nm, Sigma-Aldrich, St. Louis, MO, USA) were added as fillers into membrane surface. Humic acid (HA, Sigma-Aldrich, St. Louis, MO, USA) was used in fouling test.

### 2.2. Preparation of PSU Support and TFN Membrane

The PSU layer was synthesized using the phase inversion approach, as reported in the literature [25]. Briefly, 15 wt. % PSU was dissolved in DMF by heating (60 °C) and stirring for 5 h. PSU-DMF solution was poured onto a clean glass plate and casted to 100 µm thickness using a casting knife. The created PSU membrane was kept in DI water until use.

**Figure 1:**
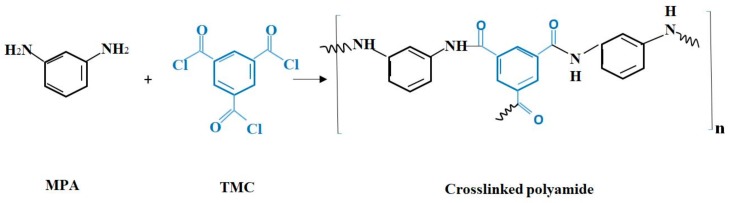
Interfacial polymerization reaction.

The interfacial polymerization was exploited to fabricate the active layer. MPD and TMC were used for this purpose. Firstly, the PSU sheet was soaked in 2 wt. % MPD aqueous solution for 1 min. Then, the resultant membrane was soaked in 0.15 wt. % TMC hexane solution for 1 min. The IP reaction is shown in Figure 1. The final product was repeatedly washed with deionized water and kept in deionized water until the test.

### 2.3. Water Flux and Salt Rejection Assessment

**Figure 2:**
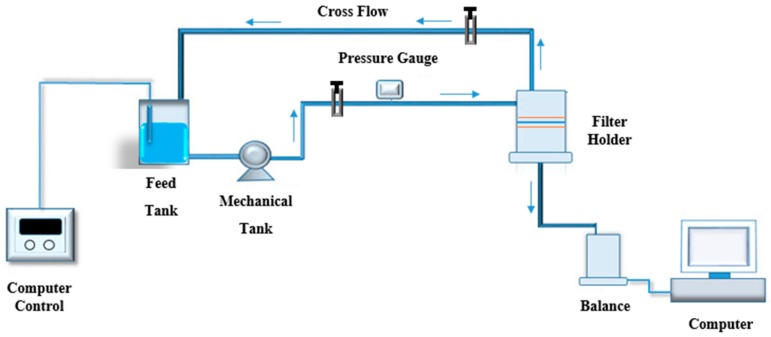
High-pressure cross-flow filtration system. Operation pressure 300 PSI and temperature 25 °C.

A high-pressure cross-flow system, as shown in Figure 2, was used to assess water flux and salt rejection. Water flux was calculated based on the following equation:

(1) $$J=\frac{V_{P}}{A\times t}$$

*J* is the water flux (L/m^2^ h), *V* the product volume (L), *A* the membrane area (m^2^), and *t* filtration time (h).

To measure the salt rejection, 2000 ppm NaCl, MgCl_2_, or Na_2_SO_4_ salts were added, in separate tests, to the feed tank. Salt rejection was calculated based on the following equation:

(2) $$R=\left(1-\frac{Cp}{Cf}\right)\times 100$$

where *Cp* and *Cf* are permeate and feed solution conductivities, respectively.

### 2.4. Organic Fouling and Bactericidal Activity Estimation

100 mg/L humic acid-water solution was used as feed solution to assess membrane fouling. The fluxes of TFC and TFN membranes were regulated to a specific value by changing the filtration pressure in order to reduce permeation drag influence on fouling extent. The antifouling performance was estimated by the following equation:

*J_n_* = *J_t_*/*J_i_* (3)

where *J_n_*, *J_t_*, and *J_i_* are the normalized flux, water flux at different time during filtration, and initial flux, respectively.

The bactericidal activity of TiO-TFN membrane was evaluated based on the literature [26]. Briefly, *Escherichia coli* bacteria cells were cultured with Luria-Bertani medium for 12 h at 37 °C and diluted with sterilized water to a specific concentration (150 µL, total 1.0 × 104 cells). The dilution was spread on 0.1 wt. % TiO_2_ TFN and TFC membranes. Then, the membranes were left in an incubator at 37 °C. 10-W bulb was used as UV light source. After a specific period of time, 1.0 wt. % sodium chloride-aqueous solution was used to collect the cells. The collected solutions were poured onto a LB-agar plate and incubated for 12 h to estimate colonies formation as a function of time.

### 2.5. Characterization Methods

The surface morphology was studied by SEM (JSM-5610LV, JEOL Ltd., Peabody, MA, USA) and operated at 20 kV. The samples were platinum-sputtered for 60 s to enhance conductivity. AFM (Seiko SPA-300 HV, Tokyo, Japan) with tapping mode in air was used to investigate surface roughness. A piece of membrane (2 × 2 µm) was tested and root mean square (RMS) was reported. Sessile drop method was used to estimate the contact angle by using video system (VCA-3000S, AST products, Inc, Billerica, MA, USA) using Milli-Q DI water (pH = 7; 4 μL droplet). Each sample was tested several times and the average was reported.

## 3. Results and Discussion

### 3.1. Contact Angle Measurements

**Figure 3:**
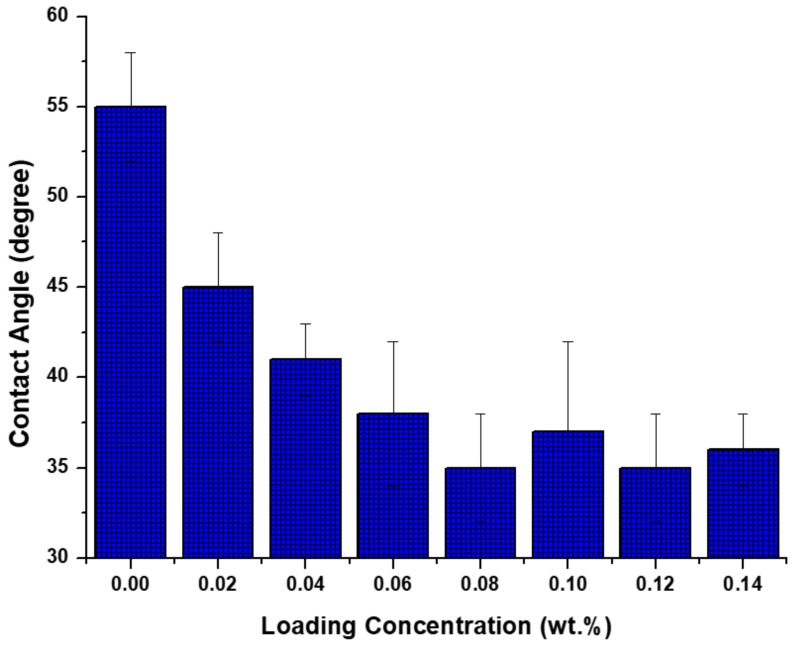
Effects of NP concentration on water contact angle.

As indicated in Figure 3, upon the addition of TiO_2_ NPs, the hydrophilic characteristic of NPs was imparted to PA layer and as a result the membrane showed lower contact angle. Another explanation based on Jun et al. [27] is that the better hydrophilicity achieved upon the addition of nano-fillers can be attributed to NPs hydration when contacting with MPD aqueous solution as well. This hydration affects the reaction between the two monomers required for PA layer preparation and as a result some of the acyl chloride group remains unreacted at membrane surface. The hydrolysis of these groups may form more carboxylic acid functional groups, thus producing high wettability and subsequently low contact angle.

### 3.2. SEM, AFM, and EDX Analyses

**Figure 4:**
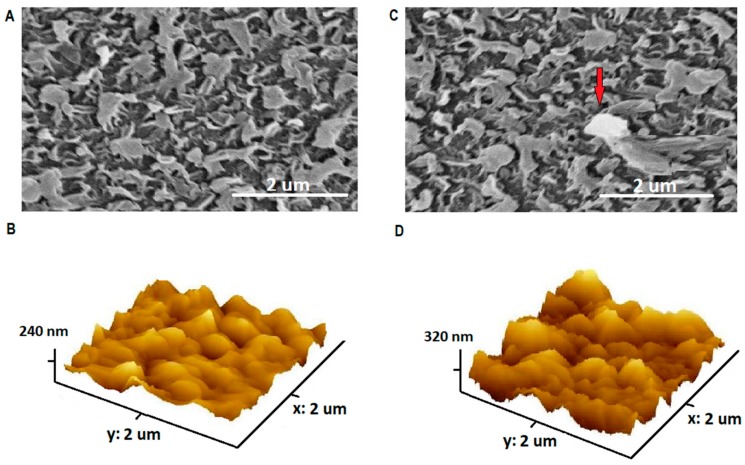
SEM Images of TFC (**A**) and 0.08 wt. TiO_2_ % TFN (**C**), and 3D AFM Images of TFC (**B**) and 0.08 wt. TiO_2_ % TFN (**D**).

The SEM and 3D-AFM images of TFC and TFN membranes are presented in Figure 4. The base membrane showed a “leaf-like” surface, consistent with the literature [27,28,29]. Partial aggregation of NPs could be observed in the membrane impregnated with high concentration of NPs, indicated by red arrow. RMS roughness was 24.6 nm for the TFC and was 44.5 nm for TFN, indicating the increase in surface roughness upon addition of NPs. The higher roughness might enhance water flux by providing a wider area for water transportation, but it could reduce fouling reduction efficiency as foulants easily adsorb on rough surfaces. The EDX analysis of TFC and TFN membranes was presented in Table 1. The surface of TFN membranes showed a higher percentage of titanium and oxygen when compared to TFC membrane, indicating the presence of TiO_2_ NPs.

### 3.3. Water Flux and Salts Rejection

**Figure 5:**
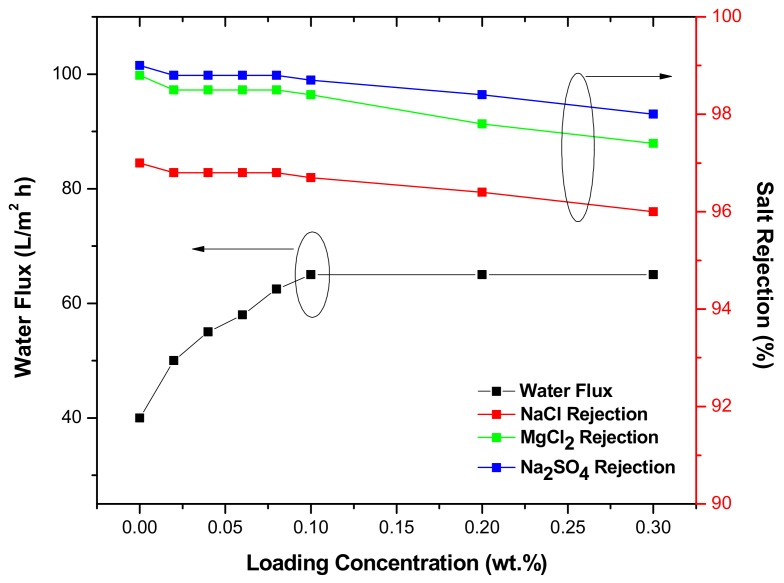
Membrane performance under 300 Psi.

Membrane performance is presented in Figure 5. By increasing TiO_2_ NPs concentration from 0 to 0.1%, the permeate flux increased from 40 to 65 L/m² h and remained constant with higher concentrations (0.2–0.3 wt. %). The better flux achieved upon addition of NPs could be caused by the improved hydrophilicity, which facilitated water molecules’ diffusion and solubilization into the membrane [30,31]. With regard to salt rejection, the rejection of NaCl, MgCl_2_, and Na_2_SO_4_ almost remained constant (>96%) by increasing the concentration of NPs from 0 to 0.1 wt. %, but decreased with higher concentrations (0.2–0.3 wt. %). The decrease in salt rejection could be ascribed to the partial aggregation of NPs in the PA layer, which likely happened in high concentrations. This aggregation could have damaged the barrier layer by generating micro gaps in PA structure. The saline water easily penetrated the membrane through these gaps; thus, reduction in water flux was observed. The salts rejection sequence was: Na_2_SO_4_ > MgCl_2_ > NaCl. This sequence can be justified based on the diffusion coefficient of salts. It is known that the molecules transfer through membranes according to the diffusion theory, and as can be seen in Table 2, the diffusion coefficient for NaCl is much higher than for Na_2_SO_4_ and for MgCl_2_; therefore, NaCl rejection was the lowest. Another justification could be the highest negative charge of SO_4_^2−^ ions [13] that was more efficiently rejected by the negatively charged membrane surface. When compared with other studies and commercial TFC membranes such as DOW-BW30 and DOW-SW30HR (Table 3), the TiO_2_ TFN membrane had higher permeability and comparable NaCl rejection.

### 3.4. Fouling Resistance and Antibacterial Efficiency

**Figure 6:**
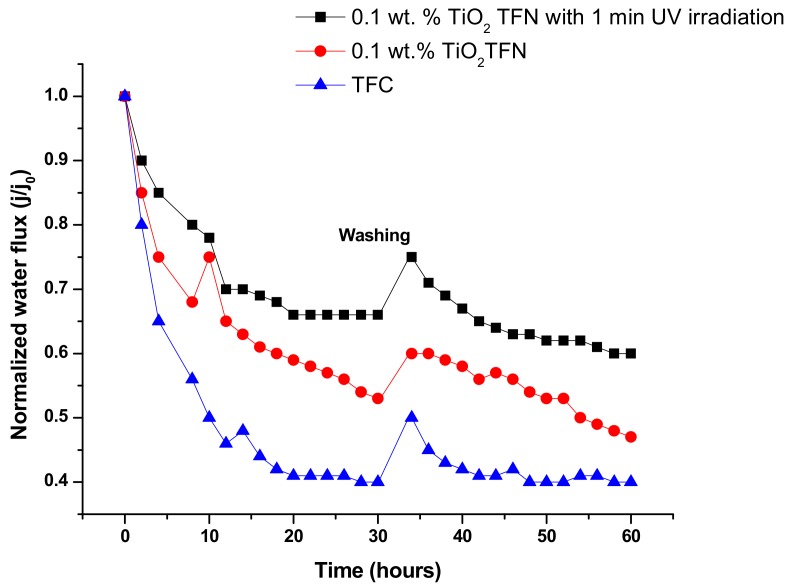
Normalized flux under HA fouling condition.

As can be observed in Figure 6, after 30 h of filtration with HA, the membrane impregnated with TiO_2_ exhibited higher water flux than the base membrane. This could be ascribed to the presence of hydrophilic TiO_2_ in the PA which reduced the attachment of foulants to membrane surface, consistent with [39,40]. For the membrane that was exposed to UV light (60 W-300 nm) for 60 s before test, better fouling resistance was achieved. It is worth mentioning that we used a short duration of irradiation (not more than 60 s), as the membrane could be degraded by longer exposure [39]. This might be due to generation of hydroxyl (OH) groups on the membrane surface, which increased the overall negative surface charge and subsequent repulsion force between the HA and PA layers. Another reason is that the generated (OH) groups could result in an increase in dissociated water adsorption on the film surface, and as a result form a compacted layer of water on membrane surface [39]. The same layer could prevent HA adsorption; thus, high fouling resistance was achieved. After the fouling test, the membranes were incubated in DI water under shaking for 30 min, and then water flux was retested again. The results indicated that the irradiated TFN reached 75% flux recovery, while the flux recovery of non-irradiated TFN and TFC were 60% and 50%, respectively. This might be due to the thicker cake layer that adsorbed on non-irradiated TFN and TFC.

**Figure 7:**
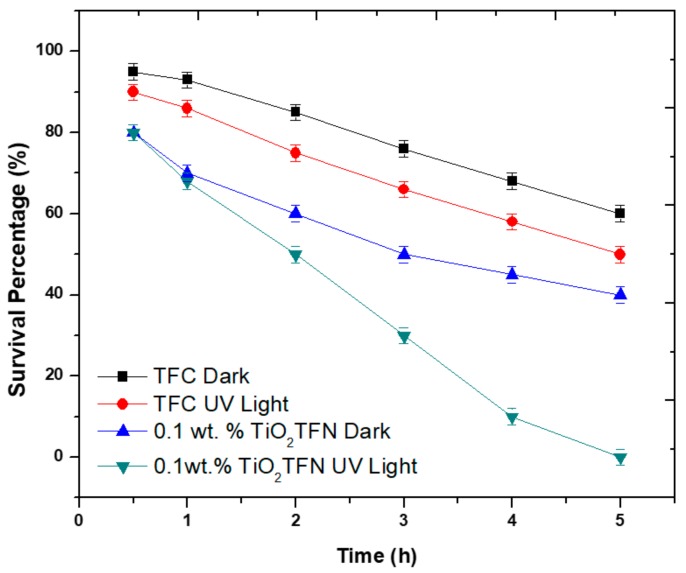
Survival of *Escherichia coli* bacterial cells in presence of TiO_2_ NPs.

To study the bactericidal activity of TiO_2_ TFN membrane, the virgin and modified membranes were immersed in *E. coli* suspension under different conditions (in dark and under UV illumination) and then the bacterial affect was investigated by counting the number of vital *E. coli* cells as a function of time. It has been indicated that using UV light with a power of 10 W does not degrade the membrane [40]. As illustrated in Figure 7, the survival ratio of bacterial cells for virgin membrane in dark decreased by 30% in 5 h; the reason behind the bacterial cells’ diminution was the insufficient nutrients during experiments. The survival ratio for TiO_2_-TFN-0.01 membrane in the dark decreased by 40% over 5 h, this could be ascribed to the significant antimicrobial properties of TiO_2_ NPs [41]. Under UV irradiation, the survival ratio reduced to 5% within 4 h, and sterilized all bacterial cells within 5 h. However, the mechanism explaining the photocatalytic death of bacterial cell is unknown, the reactive oxygen species (ROS) generation is suggested to result in the degradation of bacterial membrane and as a result cell death [42,43]. This work indicates that applying UV light on the membrane surface is crucial in minimizing the spreading and growth of bacteria.

## 4. Conclusions

TiO_2_ nanoparticles were used to modify the traditional PA TFC membrane. The modified membrane exhibited significant performance in term of water flux, organic fouling resistance, and bactericidal activity while maintaining high salt rejection. The better performance could be ascribed to (1) the high hydrophilicity and (2) good antimicrobial properties of TiO_2_ NPs. When UV light was applied, further enhancement in membrane performance was achieved. This could be ascribed to photo-generated hydrophilic carboxylic functional groups. These functional groups reduced the adherence of organic foulants and degraded bacterial cells at the membrane surface. The results presented in this work suggest that TiO_2_ can be effectively used to enhance TFC membrane performance. In addition, based on the comparison with the commercial TFN membranes, our membrane demonstrated higher water flux and comparable selectivity, indicating the possibility of practical applications of TiO_2_-TFN membrane.

## Figures and Tables

**Table 1:** EDX results on the membranes surface.

Membrane	Element (wt. %)				
	Carbon	Oxygen	Nitrogen	Titanium	Total (wt. %)
TFC	67.31	21.34	11.26	--	100
TFN	60.22	27.33	9.41	4.31	100

**Table 2:** Diffusion coefficient of salts [32].

Salt	Diffusion Coefficient (10^−9^ m^2^/s)
Na_2_SO_4_	1.23
MgCl_2_	1.25
NaCl	1.61

**Table 3:** Permeability and salt rejection of various TFN membranes.

Membrane Filler	Loading (wt. %)	Permeability (L/m^2^ h bar)	NaCl Rejection%	Ref.
Bimodal Silica	0.5 wt. %	2.58	95.7	[33]
MWCNTs	0.1 wt. %	1.75	90.0	[34]
MWCNT-TNT	0.05 wt. %	0.74	97.97	[35]
N-GOQD	0.04 wt. %	1.66	93	[36]
GO	0.015 wt. %	2.87	93.8	[13]
MCM-48	0.1 wt. %	2.12	97	[37]
Dow-SW30HR	-	1.12	98.6	[38]
Dow-BW30	-	2.15	99.4	[38]
TiO_2_	0.1 wt. %	3.14	97	This study
